# New Appliances & Things Medical

**Published:** 1908-07-11

**Authors:** 


### NEW APPLIANCES & THINGS MEDICAL.

### A NEW BED-REST.

We have lately inspected a new invalid appliance—Taylor's Patent Bed-rest—supplied by Messrs. Braun and Co., of 1 Northdown Street, King's Cross, London. This bed-rest is constructed of wood, and is intended to be fixed to the rail at the head of the bed, where it is always ready for use when wanted. By means of a weight and pulleys the rest is easily turned upwards when not in use, and remains in the vertical position at the back of the bed until required again. The invalid can himself bring the rest down into use, or return it, without getting out of bed, although this procedure is hardly to be recommended except to those approaching recovery. The special points about the Taylor Bed-rest are that the rest comes down to the patient, and can be applied to the back without moving the patient up or down in bed; that there is an ingenious shelf which can be turned down when required and adjusted in front of the invalid, for writing or reading or taking meals; and that the whole apparatus is easily attached to any bed of modern construction and is very stable. The movable shelf makes an excellent bed-table; it can be turned upwards out of the way without difficulty, and when in use it stands on the bed, clear of the patient's legs, and is well adapted for convenience in eating or reading. The parts are simply constructed and appear to be strong and durable, and we are informed that the price is low. The Taylor Bed-rest should therefore prove to be a great comfort to many invalids, while for orthopnœic patients and others who have to be propped up in bed for long periods it should be especially suitable.

